# Supplementary material for: Coupling nitrate electrochemical reduction and nitrite oxidation of ethanol for acetamide synthesis
Source: Nat Commun. 2025 Dec 27;17:1340. doi: 10.1038/s41467-025-68096-3 (PMC12873419; doi:10.1038/s41467-025-68096-3)
Supplement: Supplementary file 1 — Supplementary Information [file 41467_2025_68096_MOESM1_ESM.pdf]

Supplementary Information

Coupling Nitrate Electrochemical Reduction and Nitrite Oxidation of Ethanol for Acetamide Synthesis

Qikun Hu,[1,#] Ouwen Peng,[1,#] Jia Liu,[1,#] Mengyao Su,[1] Junyuan Feng,[2] Kun Zhang,[1] Derong Chen,[1] Zong-Xiang Xu[2] and Kian Ping Loh[1,3*]

[#]These authors contributed equally to this work.

Correspondence and requests for materials should be addressed K. P. Loh (chmlohkp@nus.edu.sg)

Address:

1 Department of Chemistry, National University of Singapore, 3 Science Drive 3, Singapore 117543, Singapore

2 Department of Chemistry, Southern University of Science and Technology, Shenzhen, 518000, China

3 Centre for Hydrogen Innovations, National University of Singapore, E8, 1 Engineering Drive 3, Singapore 117580, Singapore

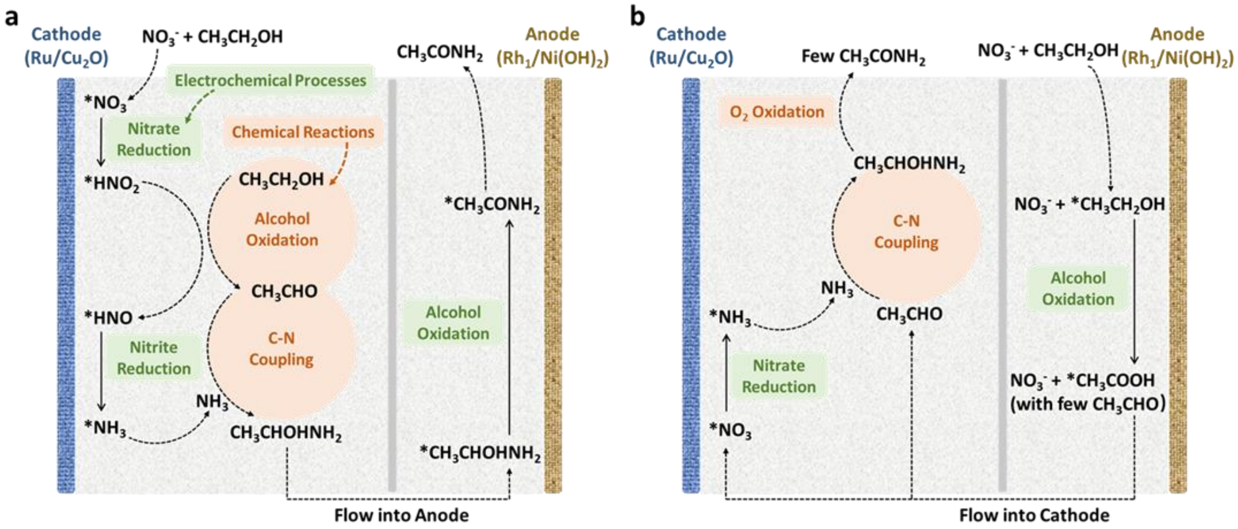

Figure S1. Full cell reaction pathway for direct acetamide electrosynthesis initiate at the anode. Much lower acetamide yield rate will be obtained.

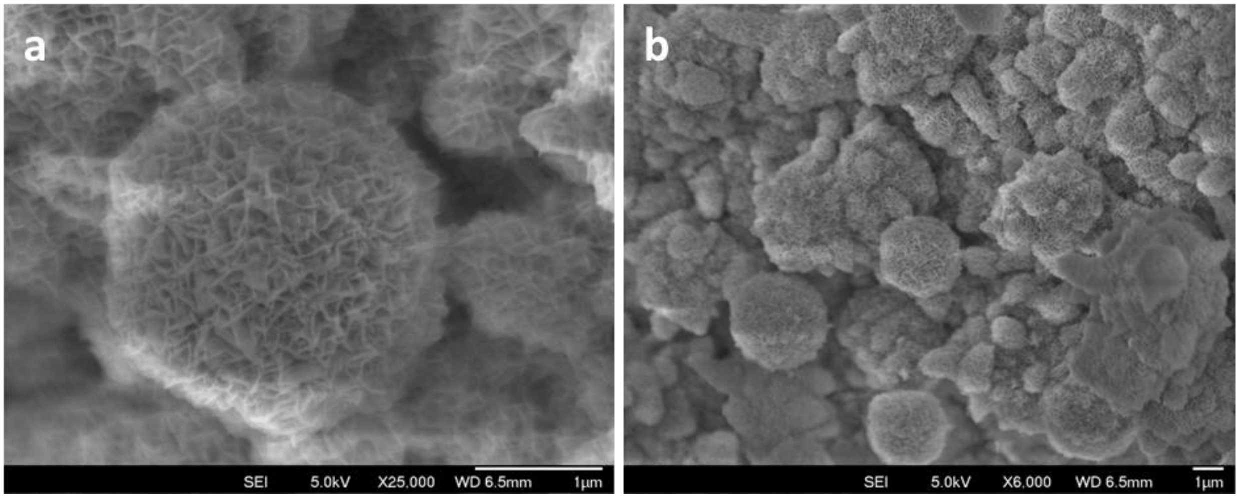

Figure S2. SEM images of $Rh_1/Ni(OH)_2$ catalyst on Ni foam at (a) 25000x and (b) 6000x magnification.

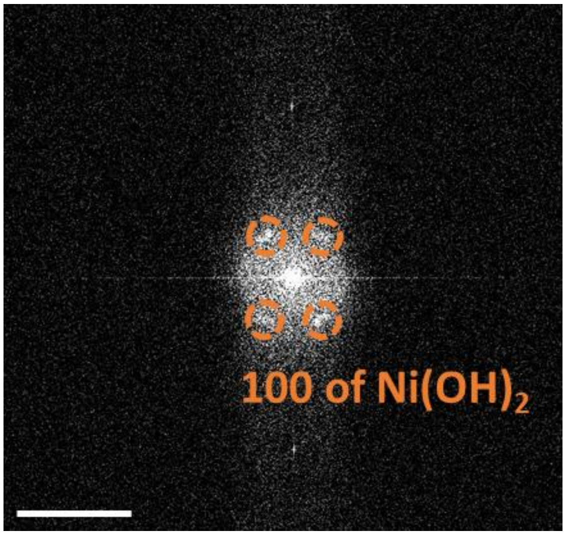

Figure S3. Fast Fourier Transform (FFT) of Figure 2a.

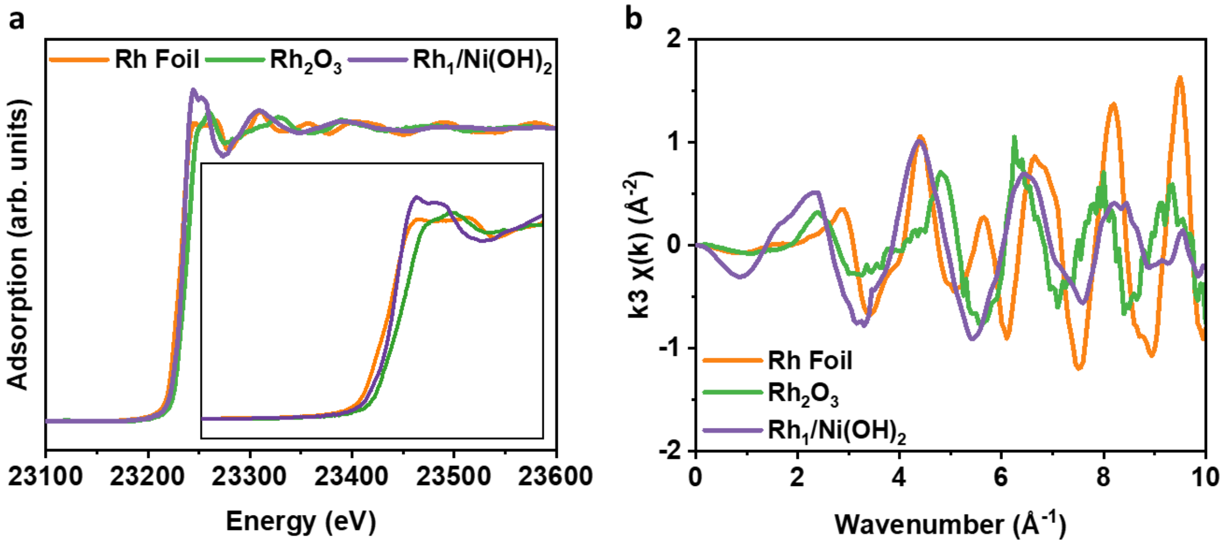

Figure S4. (a) Rh *K*-edge XANES spectra of various catalysts; (b) EXAFS for the Rh *K*-edge spectrum of $Rh_1/Ni(OH)_2$ catalyst in k space. Insert: the enlarged near-edge X-ray absorption.

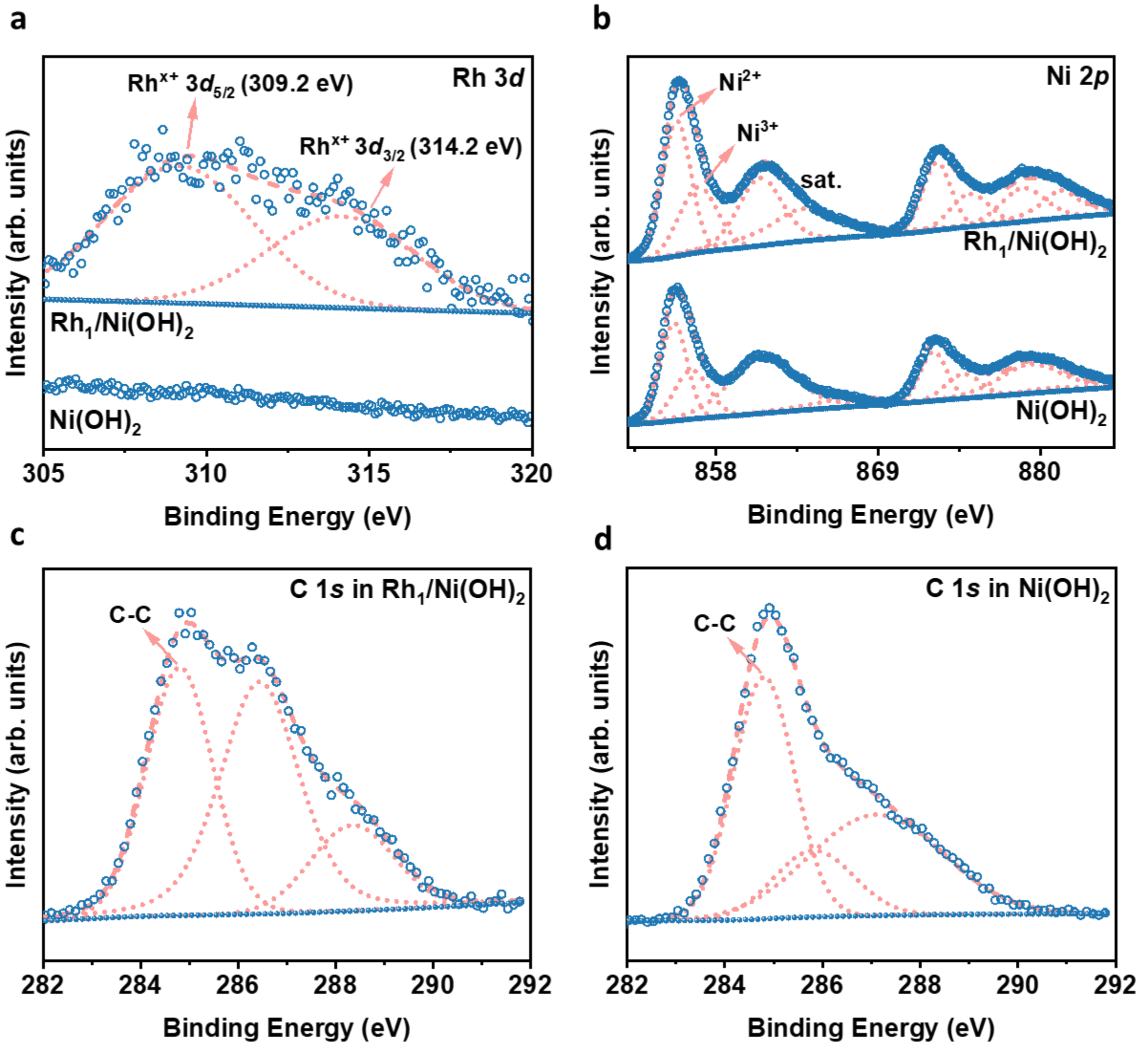

Figure S5. (a) XPS Rh_{3d} and (b) Ni_{2p} core-level spectra of $Rh_1/Ni(OH)_2$ and $Ni(OH)_2$ catalyst; C_{1s} core-level spectra in XPS of (c) $Ni(OH)_2$ and (d) $Rh_1/Ni(OH)_2$ catalysts.

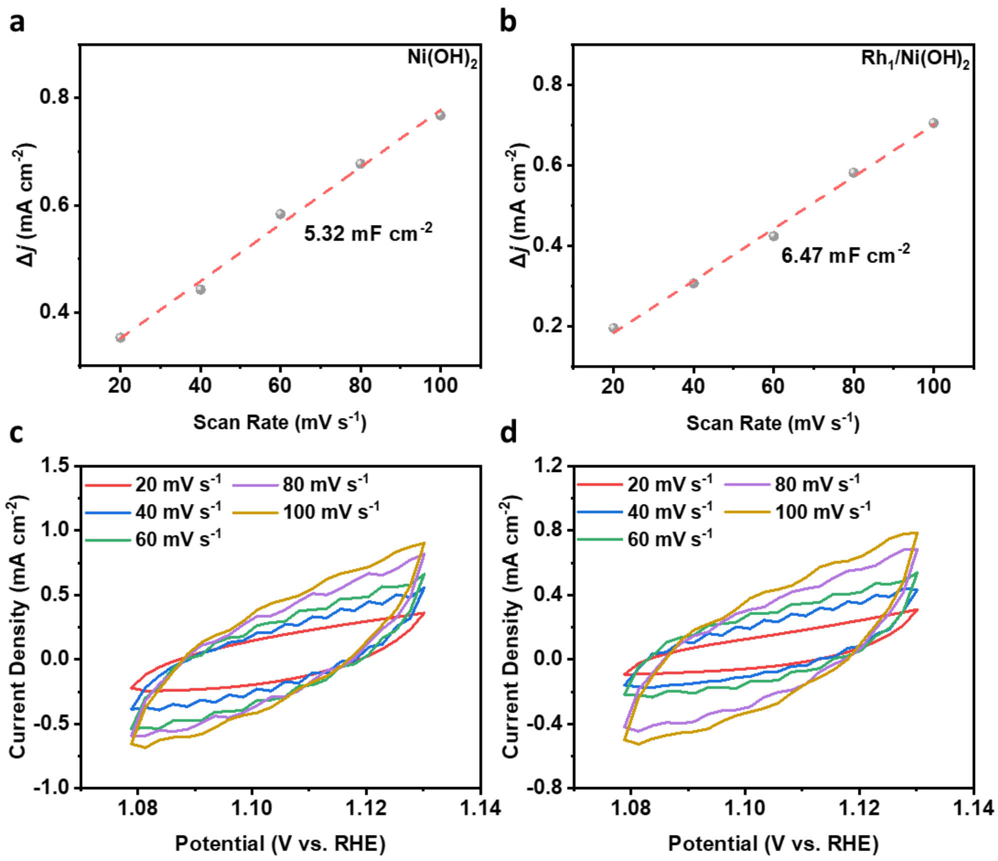

Figure S6. The linear fitting of Δ*j* (difference between anodic and cathodic current densities) versus scan rate for ECSA estimations of fully charged (a) $Ni(OH)_2$ and (b) $Rh_1/Ni(OH)_2$ catalyst; CV curves of (c) $Ni(OH)_2$ catalyst and (d) $Rh_1/Ni(OH)_2$ catalyst for ECSA estimations at various sweep rates in 1 M KOH.

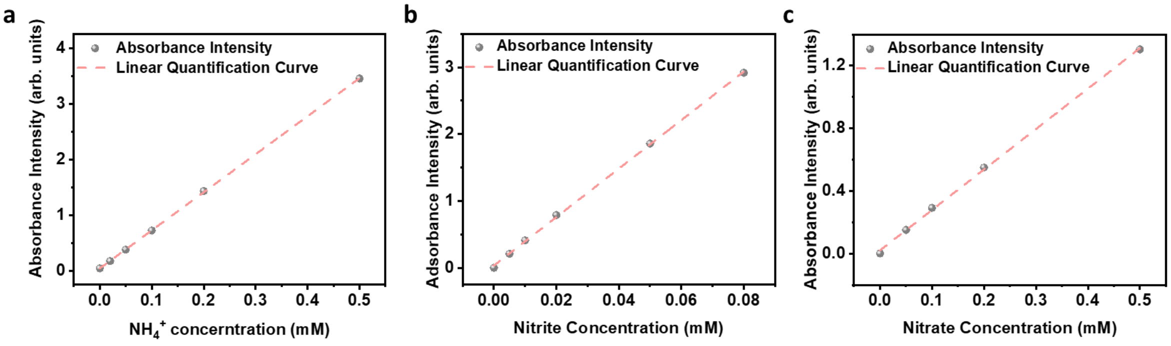

Figure S7. (a) Standard calibration curves of NH_4^+ by indophenol blue colorimetry in UV-vis (R^2 = 0.9999); (b) Standard calibration curves of nitrite by diazotization colourimetry in UV-vis (R^2 = 0.9996); (c) Standard calibration curves of nitrate in UV-vis (R^2 = 0.9992).

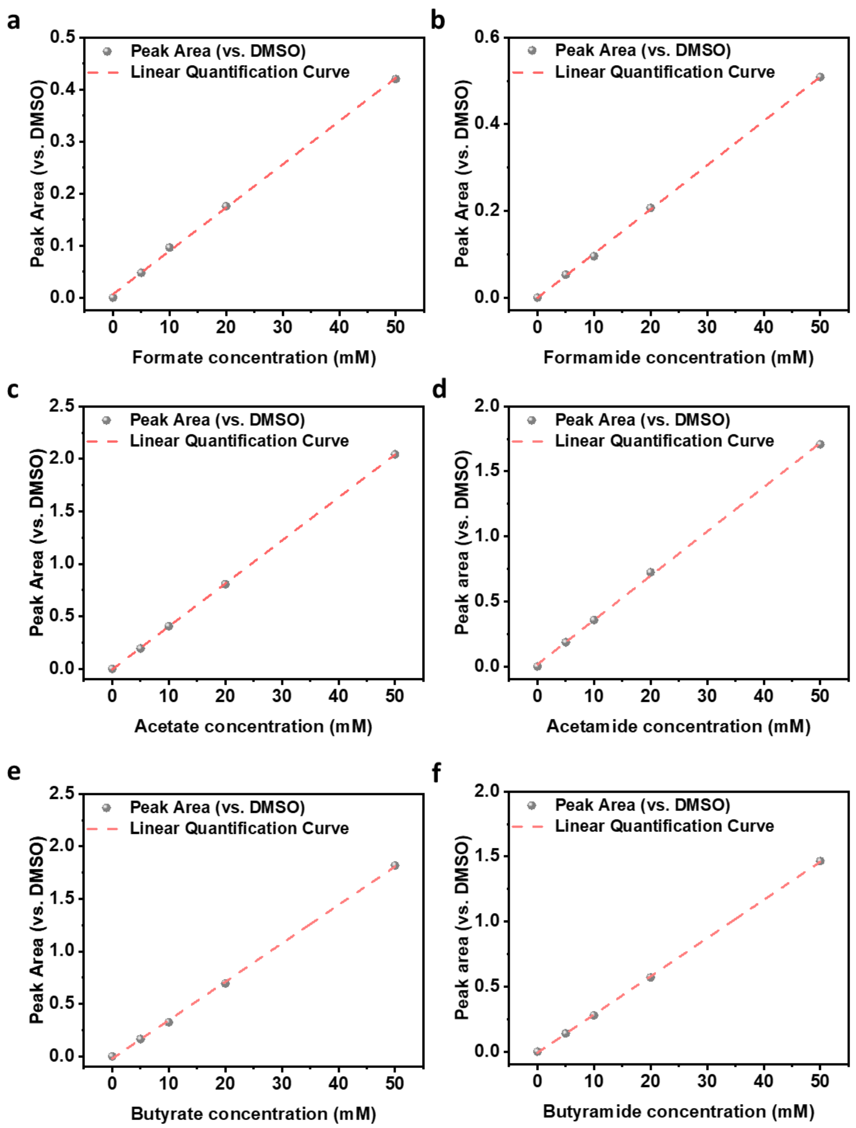

Figure S8. (a) Standard calibration curves of formate in ^{1}H NMR (R^2 = 0.9991); (b) Standard calibration curves of formamide in ^{1}H NMR (R^2 = 0.9996); (c) Standard calibration curves of acetate in ^{1}H NMR (R^2 = 0.9999); (d) Standard calibration curves of acetamide in ^{1}H NMR (R^2 = 0.9994); (e) Standard calibration curves of butyrate in ^{1}H NMR (R^2 = 0.9997); (f) Standard calibration curves of butyramide in ^{1}H NMR (R^2 = 0.9991).

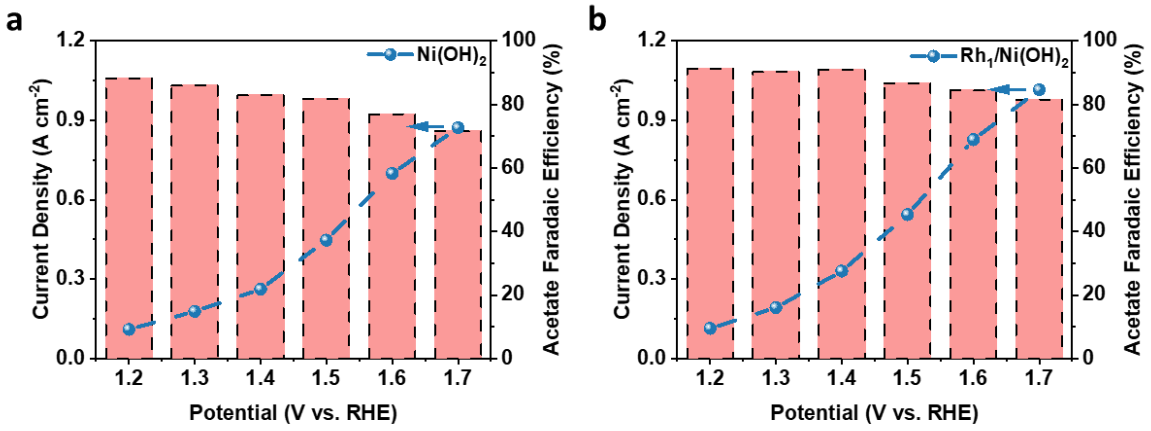

Figure S9. Current density and acetate Faradaic efficiency of (a) $Ni(OH)_2$ and (b) $Rh_1/Ni(OH)_2$ catalyst in 1 M KOH and 1 M CH_3CH_2OH water solution in H-cell.

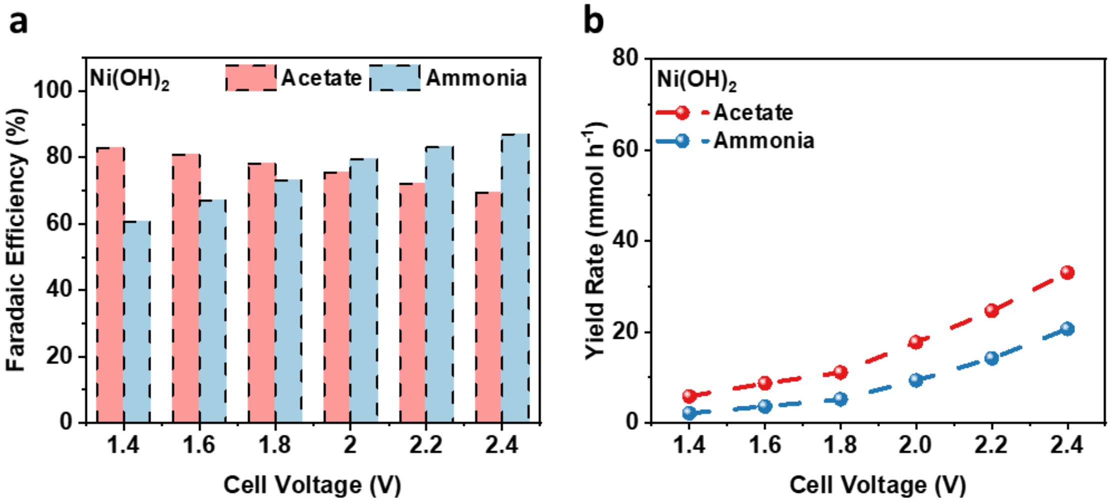

Figure S10. Acetate and ammonia (a) Faradaic efficiency and (b) yield rate of $Ni(OH)_2$ catalyst in 1 M KOH and 1 M CH_3CH_2OH water solution in the 16 cm^2 flow electrolyzer.

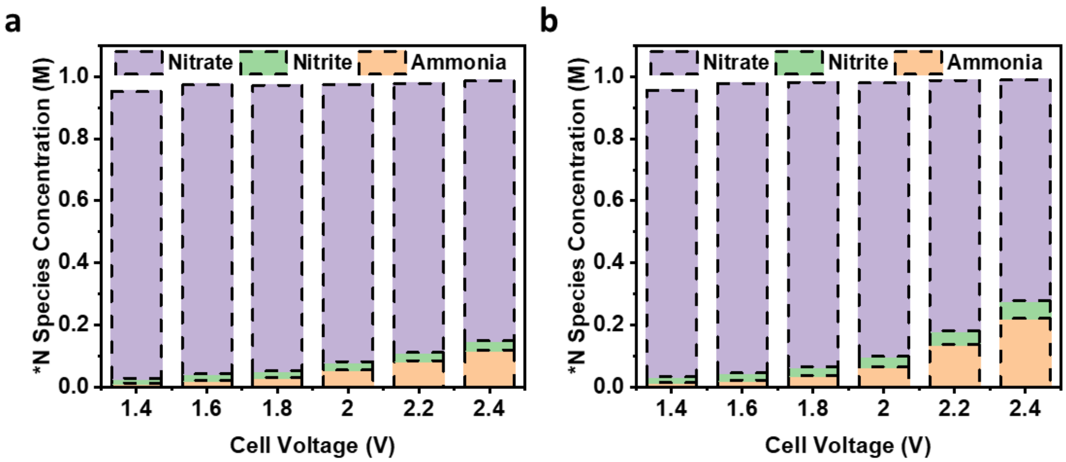

Figure S11. The concentrations of N-containing species of coupling nitrate reduction and ethanol oxidation in parallel in the 16 cm^2 flow electrolyser applying (a) $Ni(OH)_2$ catalyst and (b) $Rh_1/Ni(OH)_2$ catalyst as anode. The total concentration of N-containing species kept constant during whole reaction.

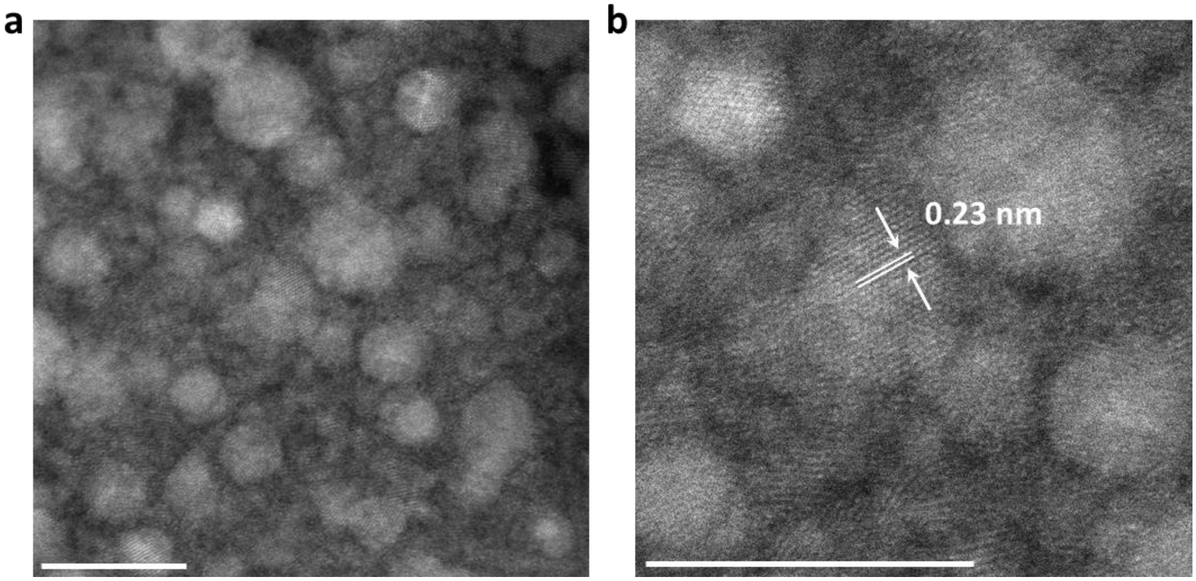

Figure S12. HRTEM images of Rh Nanoparticles/ $Ni(OH)_2$ catalyst at a higher Rh metal loading of 1 wt%, showing the presence of Rh nanoparticles (a) and (b) lattice fringes of (111) facet as the major exposed plane of Ru nanoparticles. Scale bar: 10 nm.

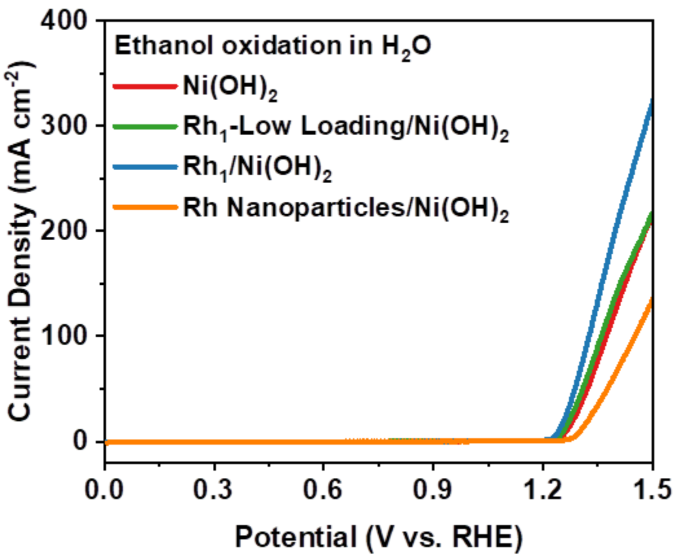

Figure S13. LSV curves of catalysts in 1 M KOH and 1 M CH_3CH_2OH water solution. $Rh_1/Ni(OH)_2$ catalyst exhibits the highest current density.

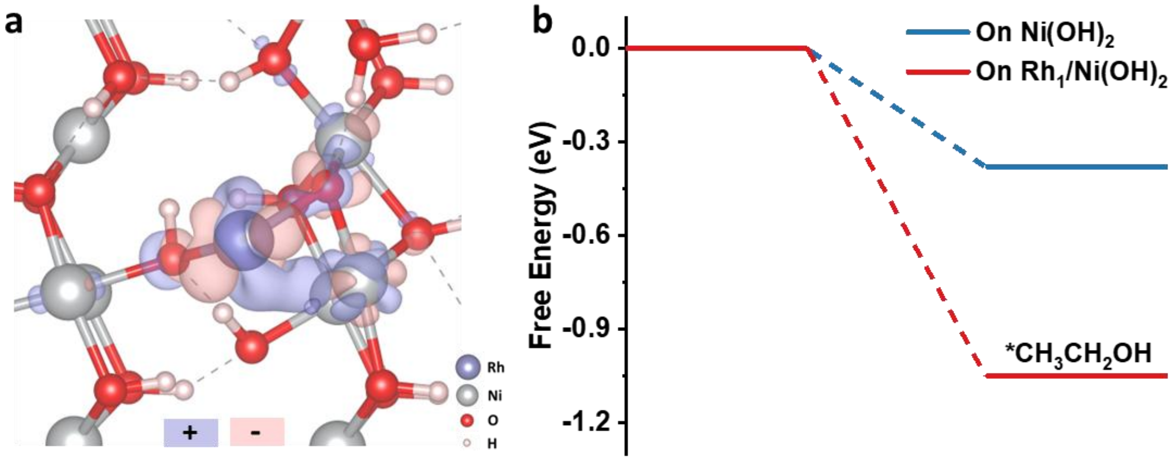

Figure S14. (a) Charge density difference of $Rh_1/Ni(OH)_2$ catalyst; (b) Free energy for CH_3CH_2OH adsorption on $Ni(OH)_2$ and $Rh_1/Ni(OH)_2$ catalyst.

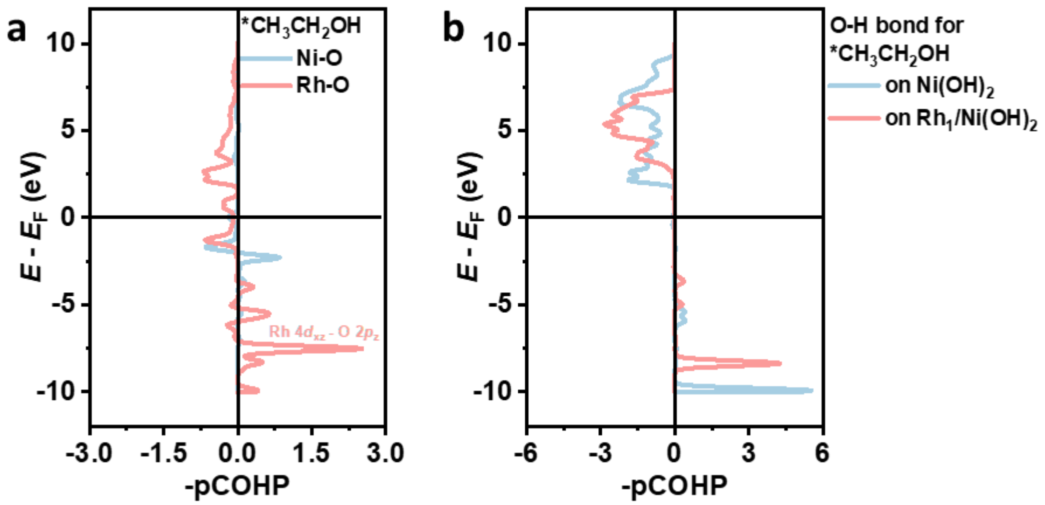

Figure S15. (a) pCOHP evaluation for CH_3CH_2OH adsorption on $Ni(OH)_2$ and $Rh_1/Ni(OH)_2$ catalyst; (b) pCOHP of O-H bond in adsorbed CH_3CH_2OH on $Ni(OH)_2$ and $Rh_1/Ni(OH)_2$ surface.

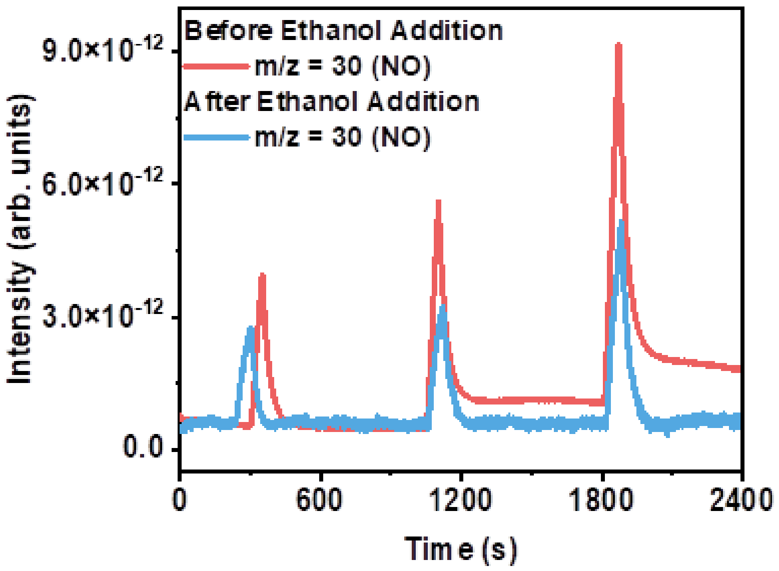

Figure S16. DEMS validation of NO intermediate (m/z =30) generation for NO_3RR with ethanol.

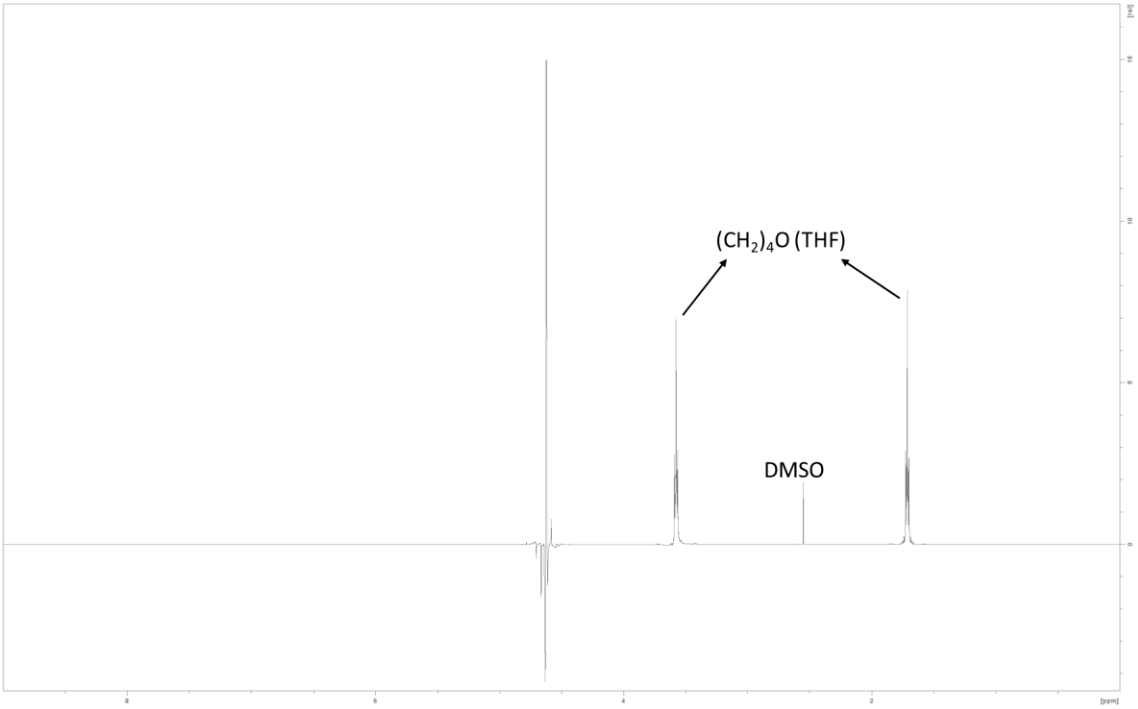

Figure S17. ^{1}H NMR validation of the electrolyte before THF oxidation by nitrite at cathode.

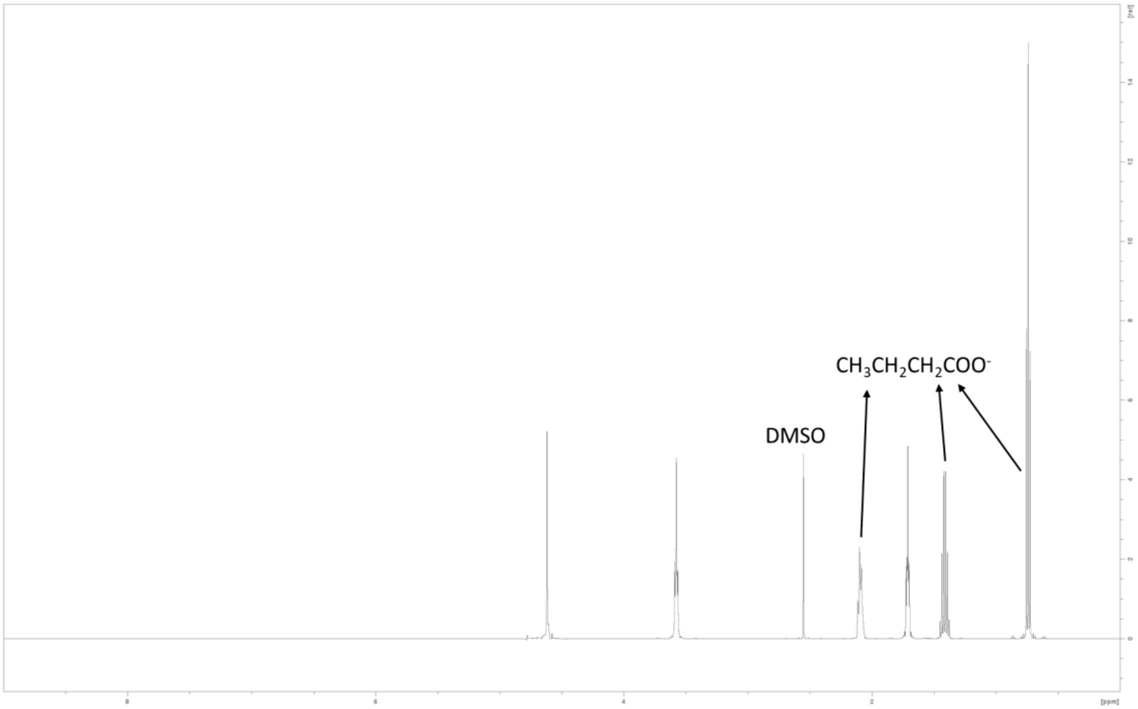

Figure S18. ^{1}H NMR validation of the electrolyte after THF oxidation by nitrite at cathode. Butanoate was obtained as final product.

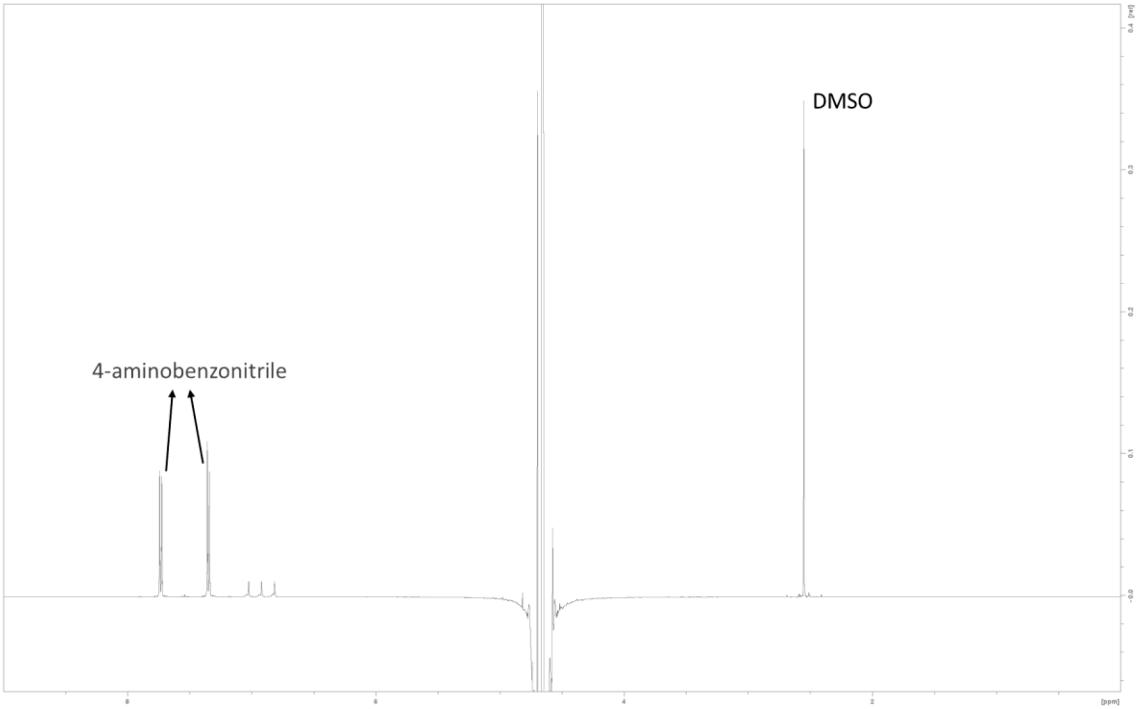

Figure S19. ^{1}H NMR validation of the electrolyte before 4-aminobenzonitrile oxidation by nitrite at cathode.

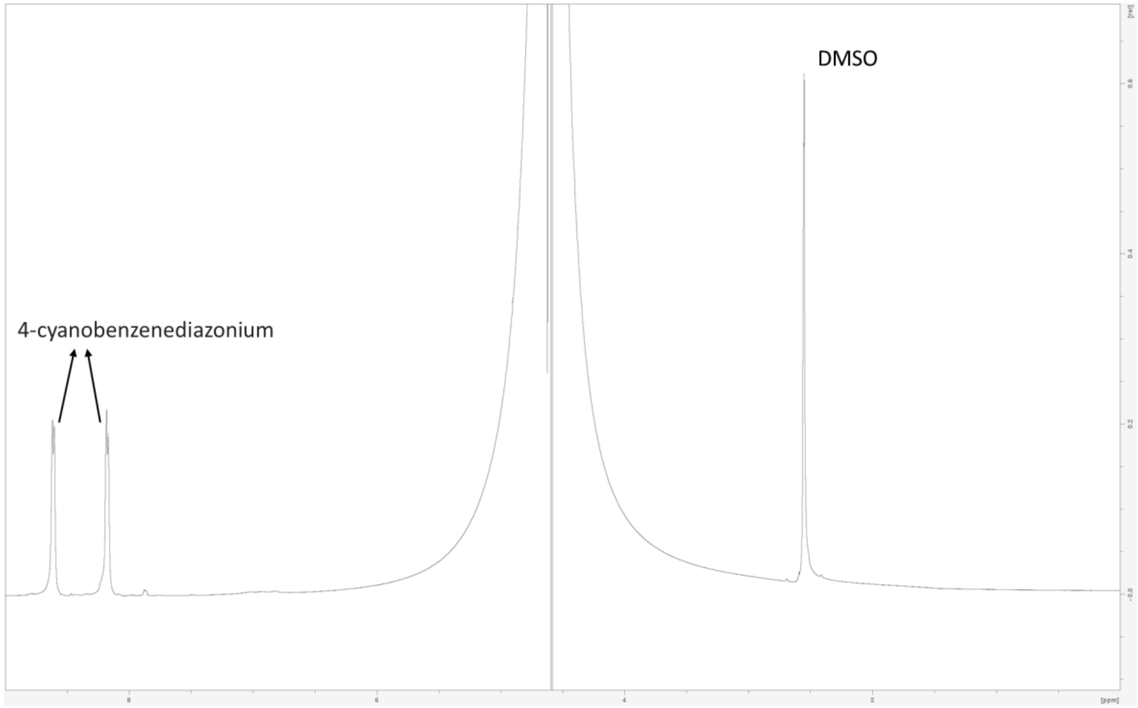

Figure S20. ^{1}H NMR validation of the electrolyte after 4-aminobenzonitrile oxidation by nitrite at cathode. 4-cyanobenzenediazonium was obtained as final product.

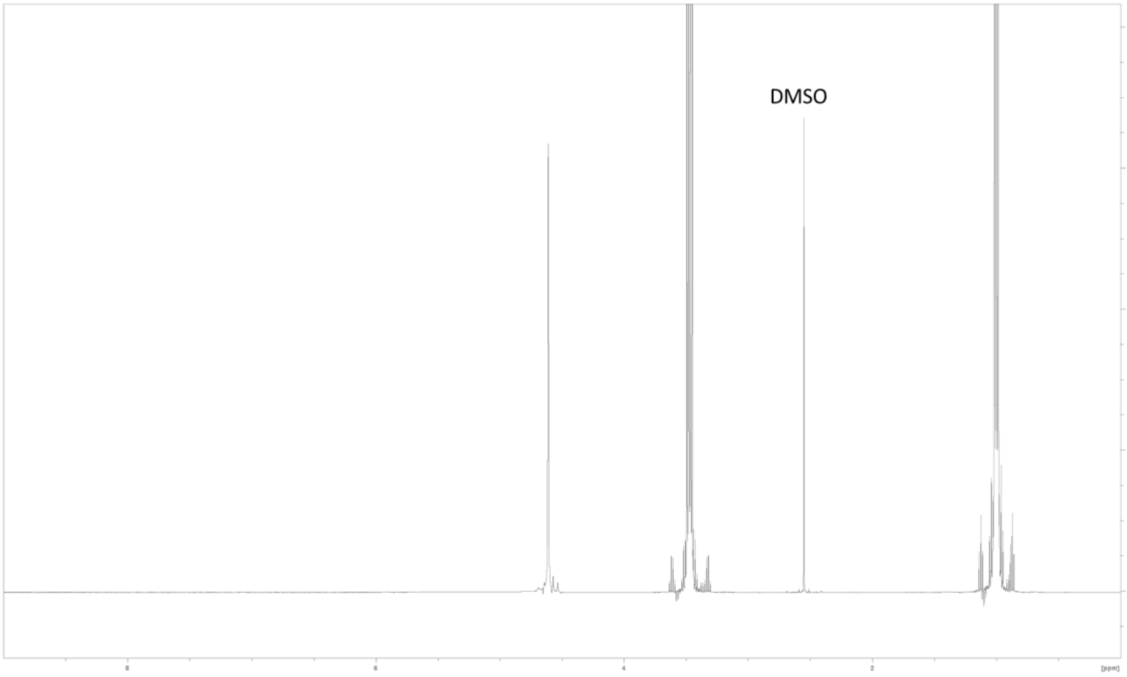

Figure S21. ^{1}H NMR validation of mixing 0.1 M KOH, 0.05 M $(NH_4)_2CO_3$ and 1 M CH_3CH_2OH in water solution. No α-aminoethanol product was found in the spectrum.

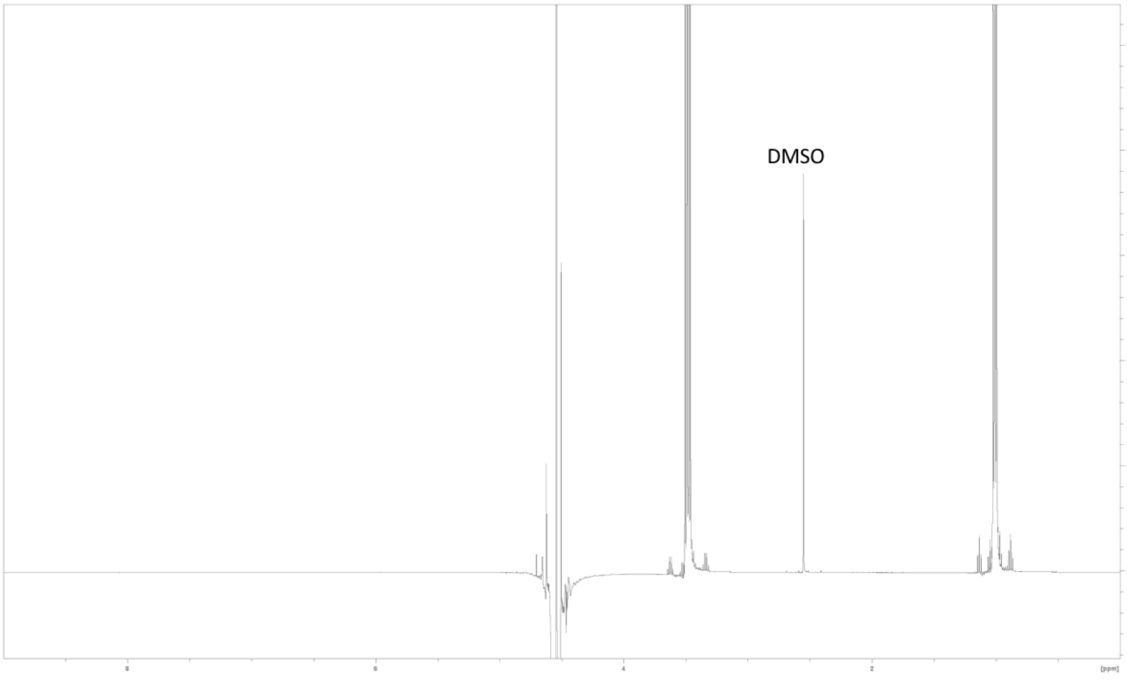

Figure S22. ^{1}H NMR validation of mixing 0.1 M KOH, 0.1 M KNO_2 and 1 M CH_3CH_2OH in water solution. No α-aminoethanol product was found in the spectrum.

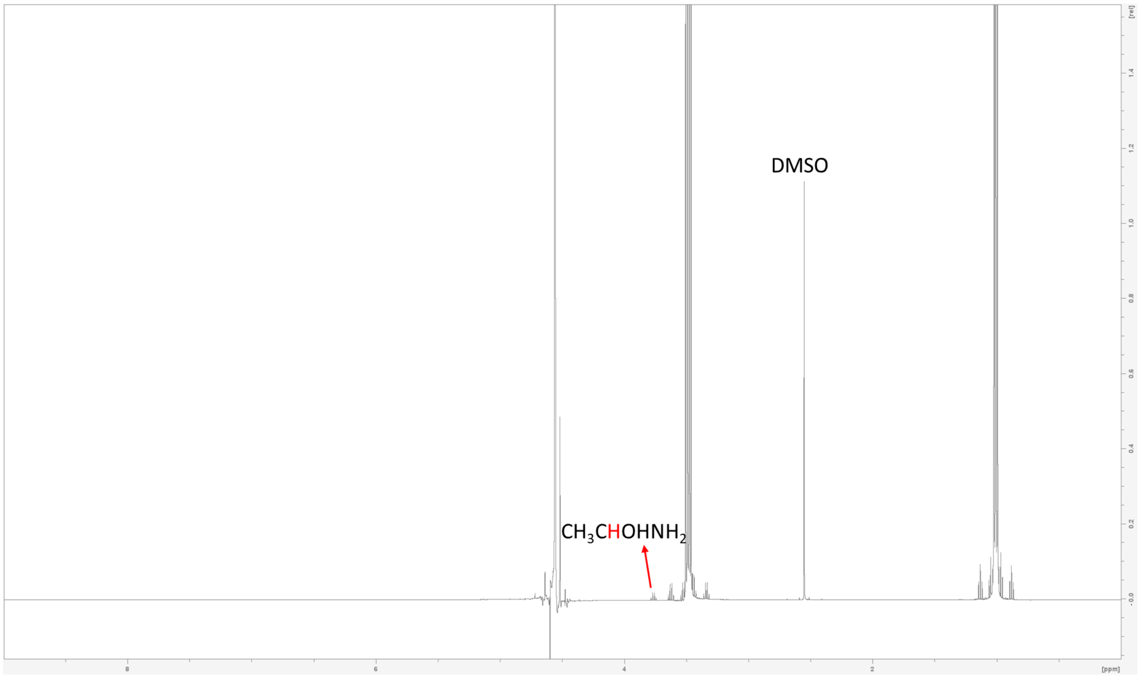

Figure S23. ^{1}H NMR validation of mixing 0.1 M KOH, 0.1 M KNO_2, 0.05 M $(NH_4)_2CO_3$ and 1 M CH_3CH_2OH in water solution. α-aminoethanol product can be found in the spectrum.

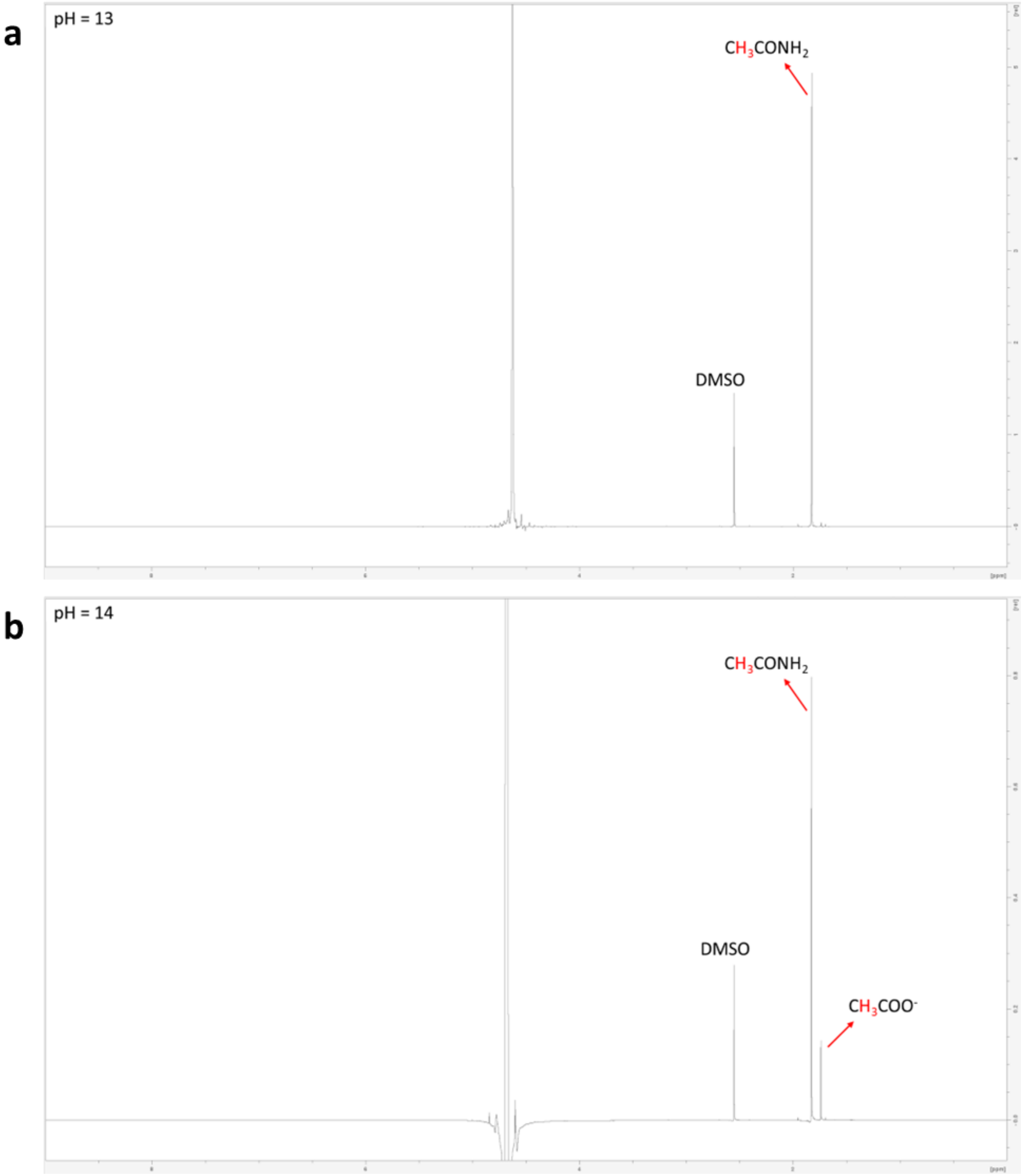

Figure S24. ^{1}H NMR validation of acetamide stability in aqueous solution with pH (a) 13 and (b) 14. If pH is 14, acetamide will gradually decompose into acetate and ammonia.

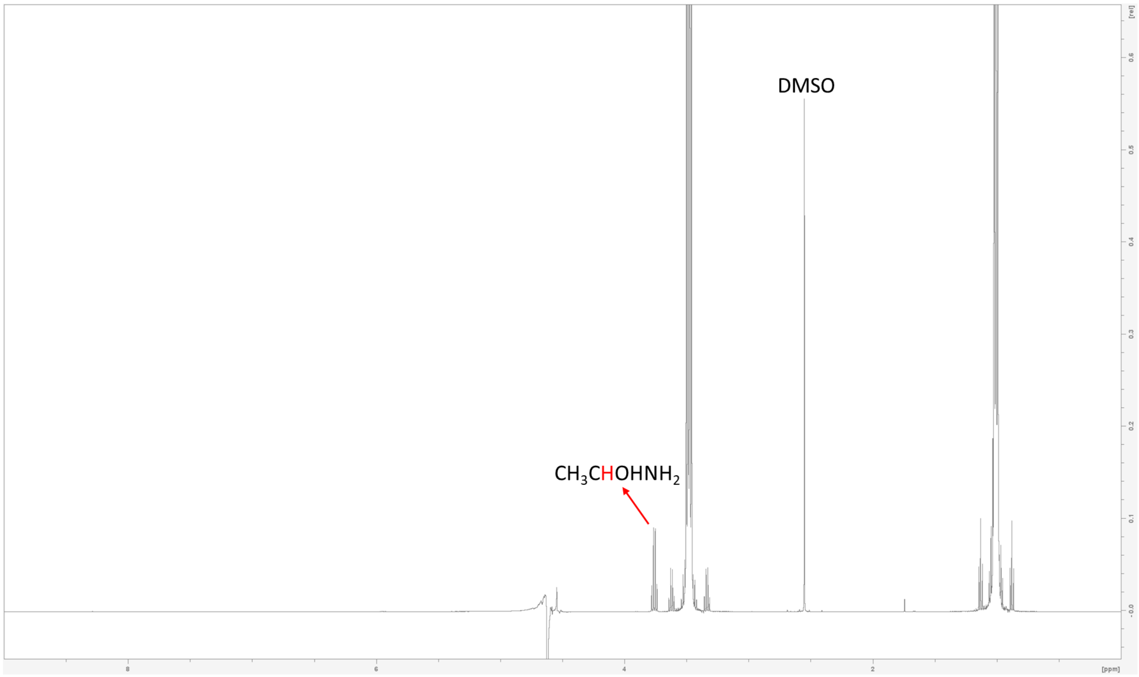

Figure S25. ^{1}H NMR validation of cathode electrolyte from the 16 cm^2 flow electrolyzer under 2.4 V cell voltage. α-aminoethanol product can be found in the spectrum.

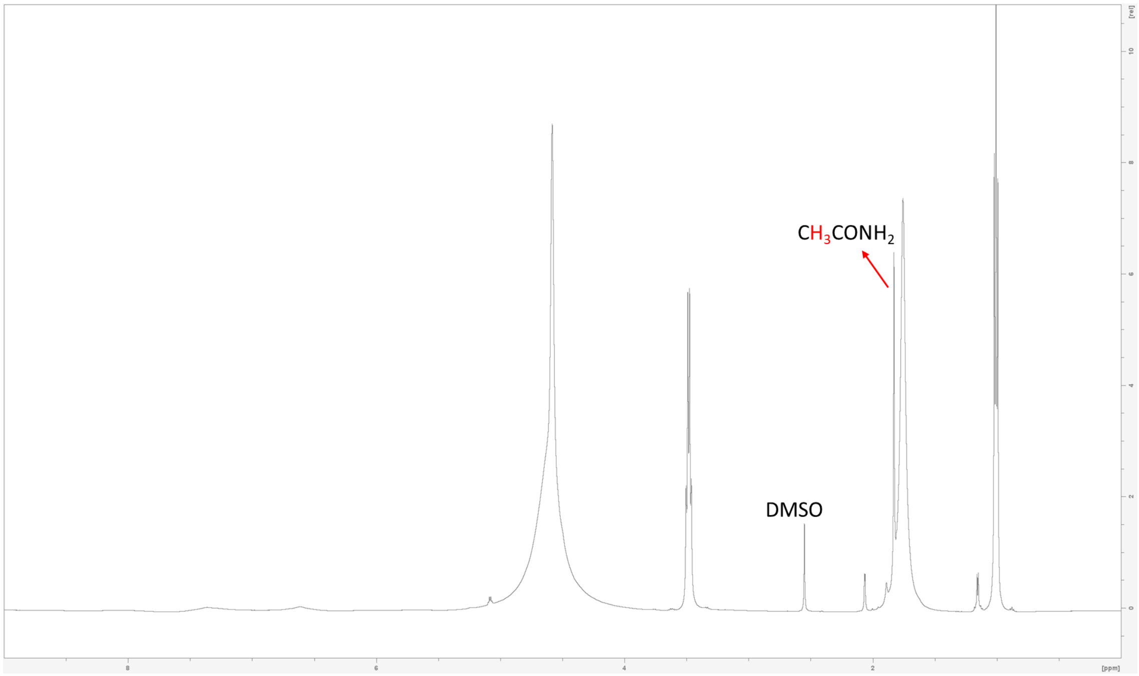

Figure S26. ^{1}H NMR validation of synthesized acetamide (CH_3CONH_2) from electrolyte under 2.4 V cell voltage. It has same chemical shift with standard CH_3CONH_2.

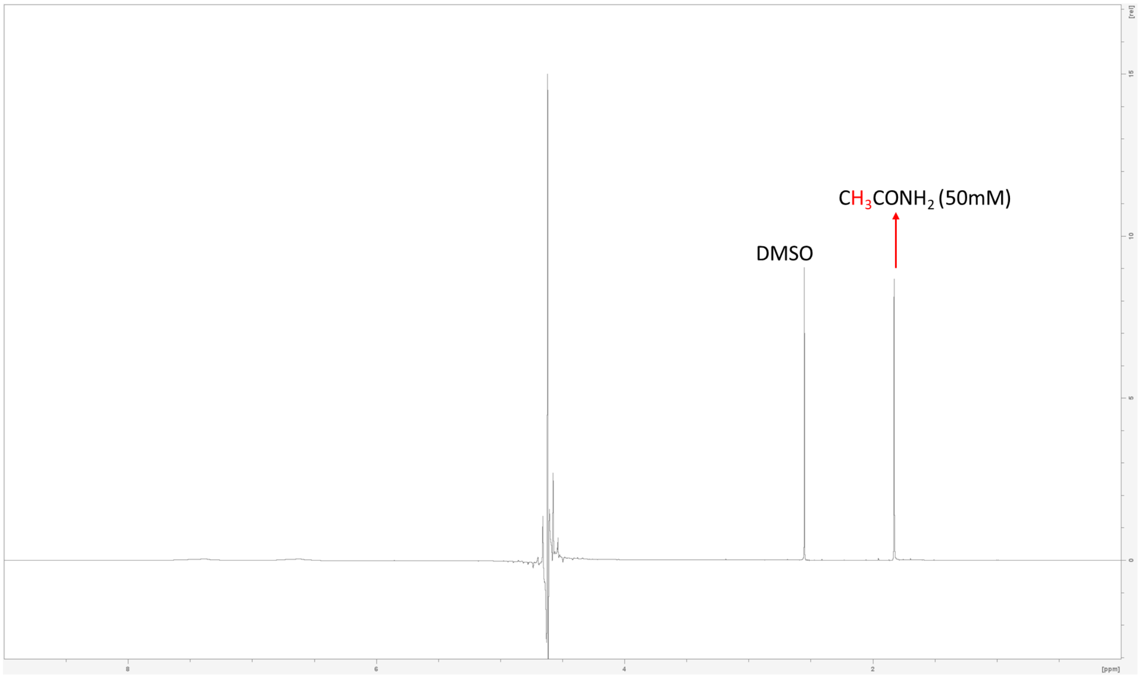

Figure S27. ^{1}H NMR validation of standard formamide acetamide (CH_3CONH_2) from solution used for quantitative determination.

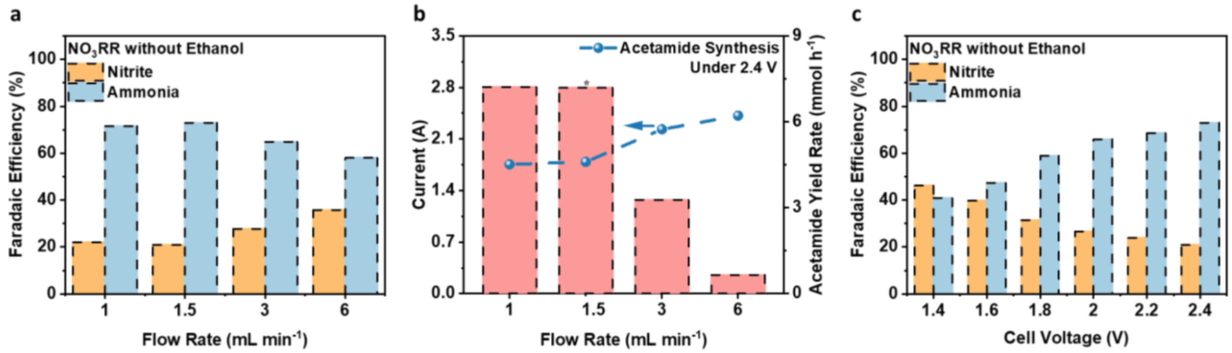

Figure S28. (a) Nitrite and ammonia and (b) acetamide synthesis performance at different flow rates (mL min^{-1}) for 16 cm^2 flow electrolyzer under 2.4 V cell voltage. 1.5 mL min^{-1} is found to be the most suitable flow rate for acetamide synthesis. (c) Nitrite and ammonia Faradaic efficiency under different cell voltage for 16 cm^2 flow electrolyzer at 1.5 mL min^{-1} flow rate.

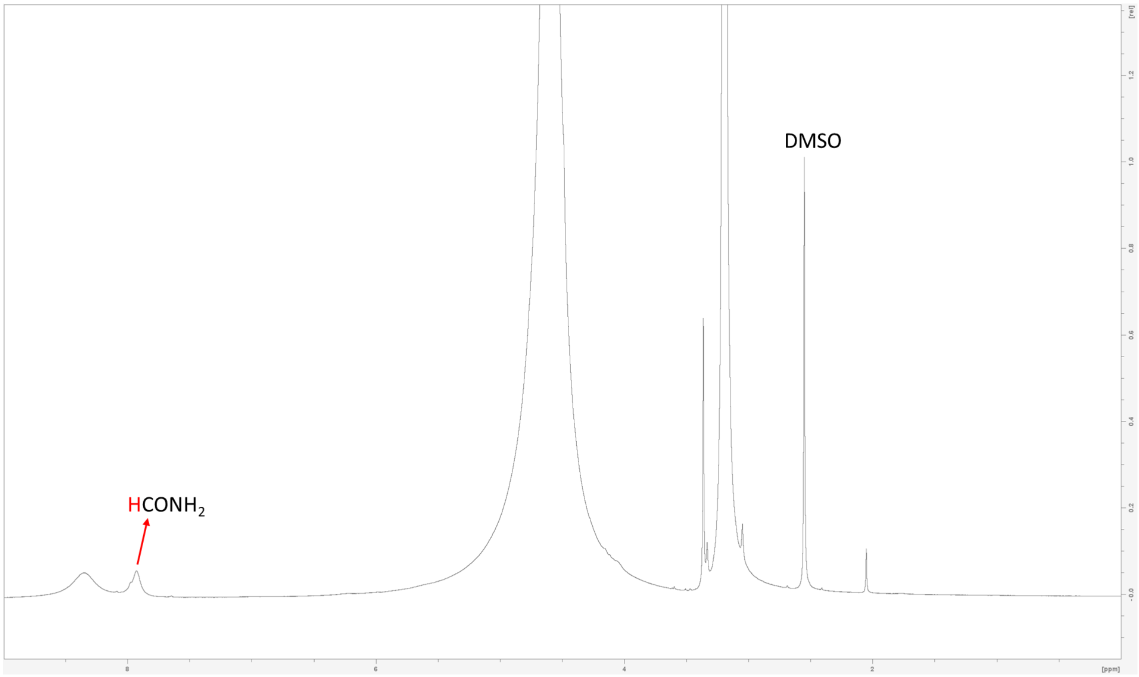

Figure S29. ^{1}H NMR validation of synthesized formamide ($HCONH_2$) from electrolyte under 2.4 V cell voltage. It has same chemical shift with standard $HCONH_2$.

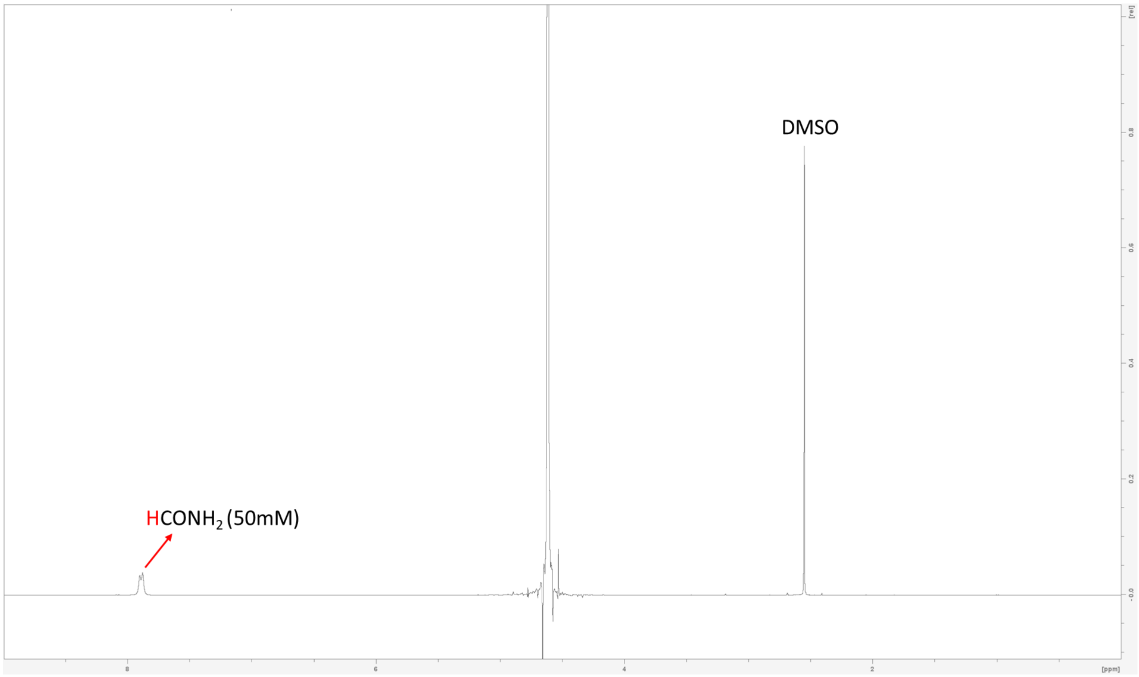

Figure S30. ^{1}H NMR validation of standard formamide ($HCONH_2$) from solution used for quantitative determination.

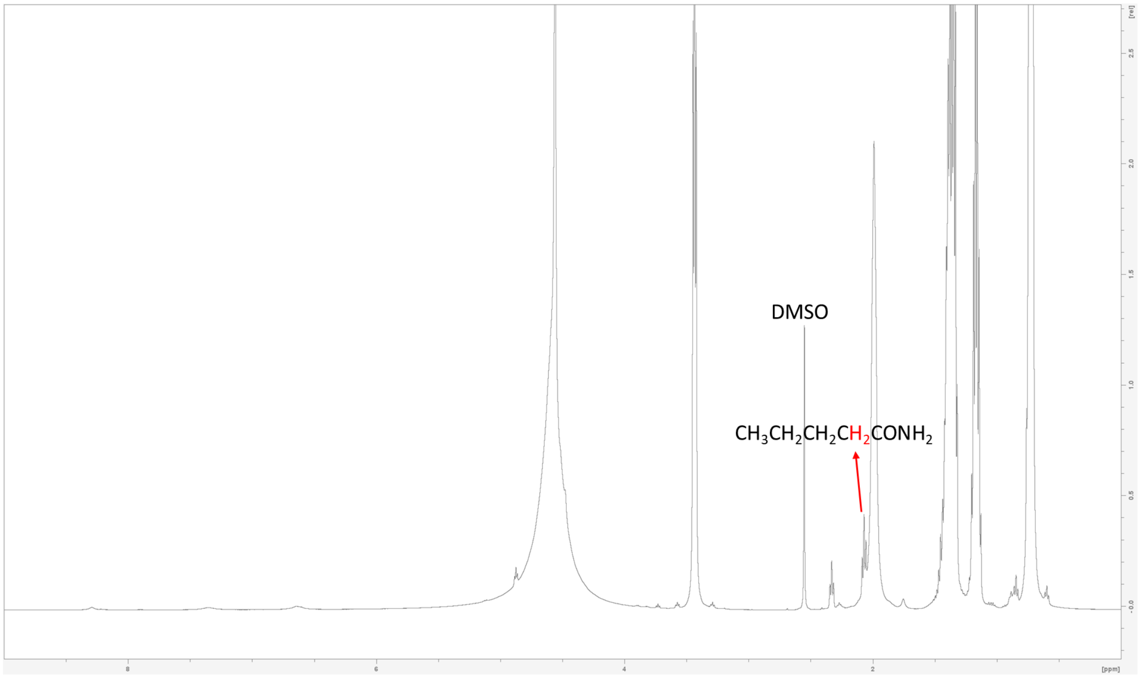

Figure S31. ^{1}H NMR validation of synthesized butyramide ($CH_3CH_2CH_2CH_2CONH_2$) from electrolyte under 2.4 V cell voltage. It has same chemical shift with standard $CH_3CH_2CH_2CH_2CONH_2$.

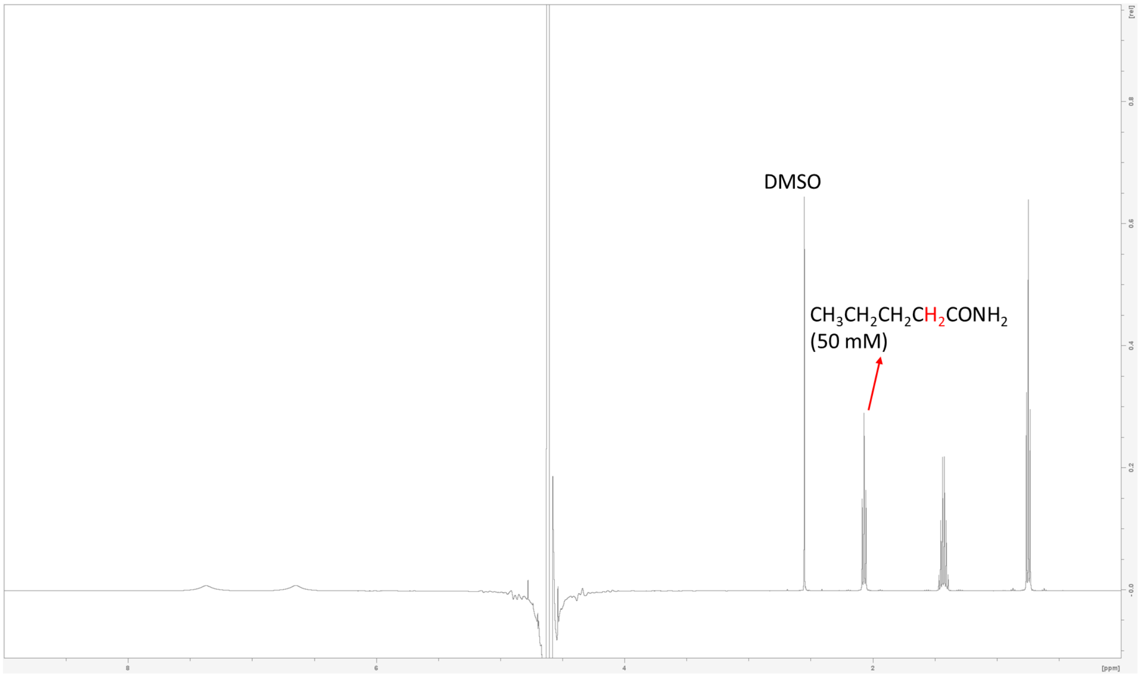

Figure S32. ^{1}H NMR validation of standard formamide butyramide ($CH_3CH_2CH_2CH_2CONH_2$) from solution used for quantitative determination.

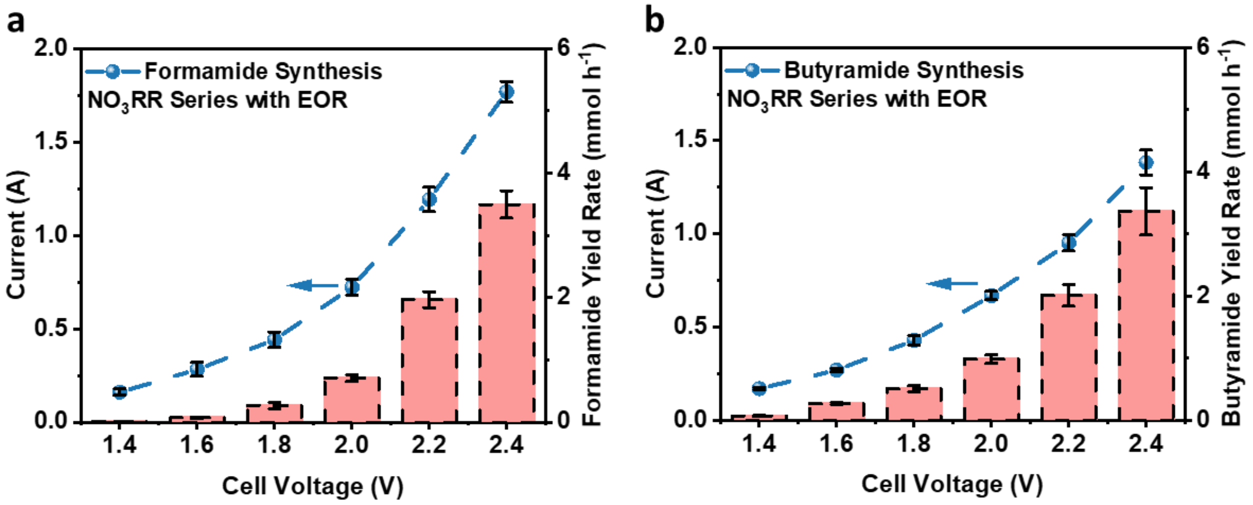

Figure S33. Current and yield rate for (a) formamide (b) butyramide electrosynthesis. Error bars (in standard deviation) are present for 3 repetitive experiments.

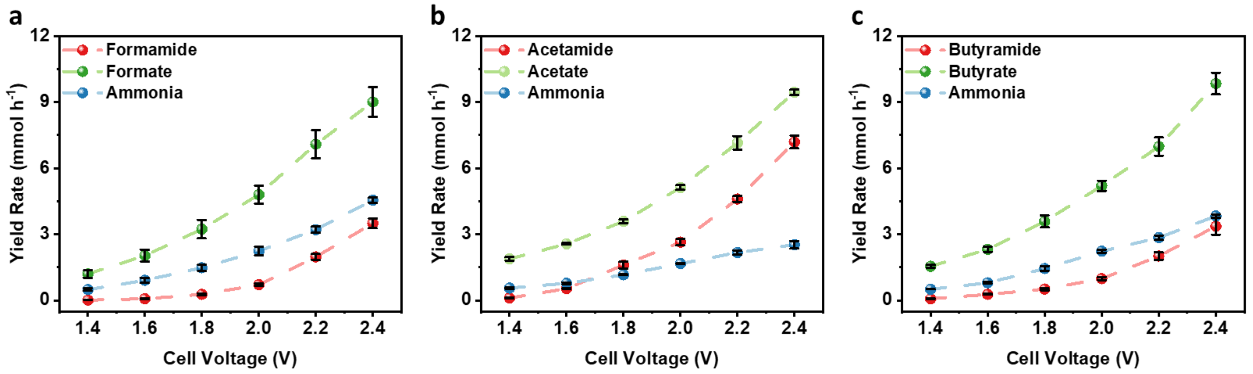

Figure S34. Products yield rate during direct (a) formamide, (b) acetamide and (c) butyramide synthesis in the 16 cm^2 flow electrolyzer. Error bars (in standard deviation) are present for 3 repetitive experiments.

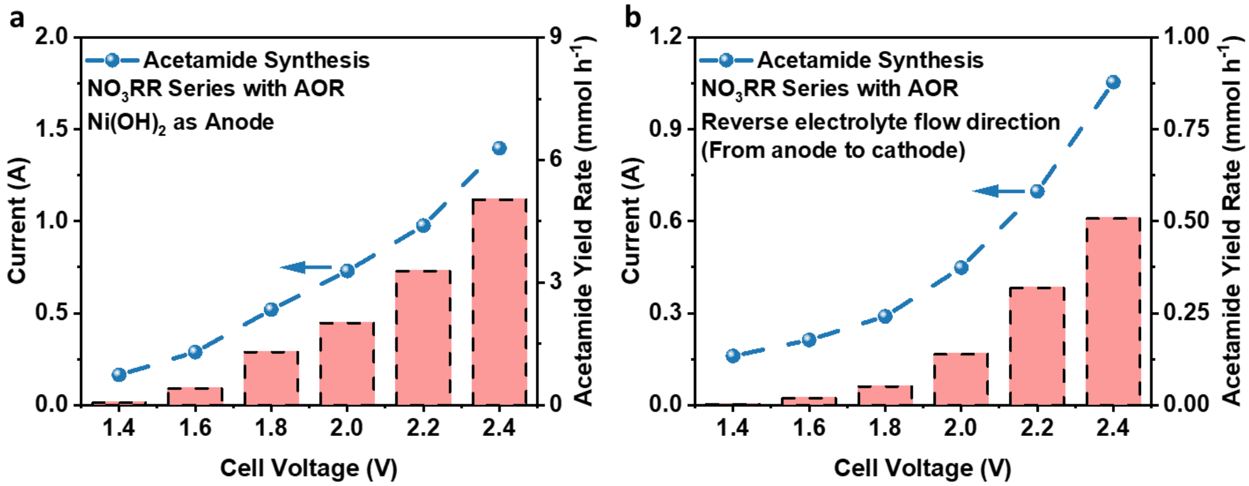

Figure S35. (a) Acetamide synthesis performance by using bulk $Ni(OH)_2$ as anode; (b) Acetamide synthesis performance at reverse electrolyte flow (from anode to cathode, without circulation) in 16 cm^2 flow electrolyzer. Although the electrolyte was the same, acetamide yield rate decreases a lot.

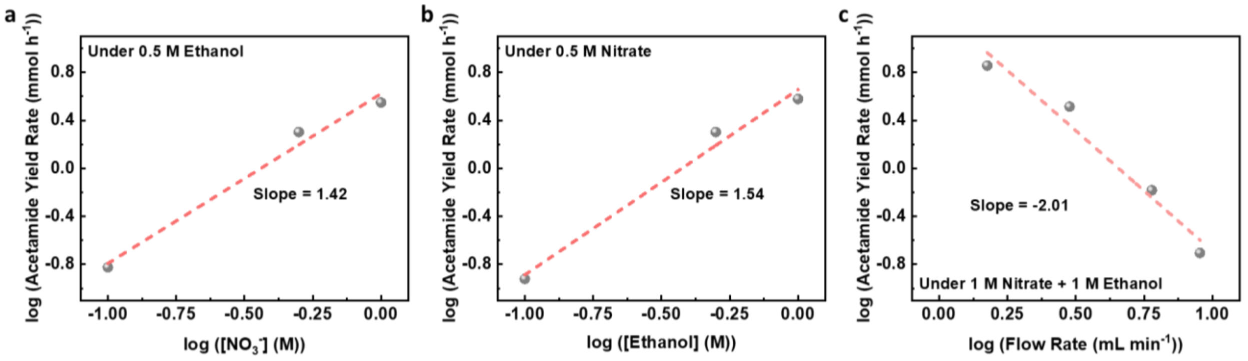

Figure S36. Logarithm of the acetamide yield rate as a function of the logarithm of the (a) concentration of nitrate, (b) concentration of ethanol and (c) flow rate 16 cm^2 in 16 cm^2 flow electrolyzer under 2.4 V cell voltage.

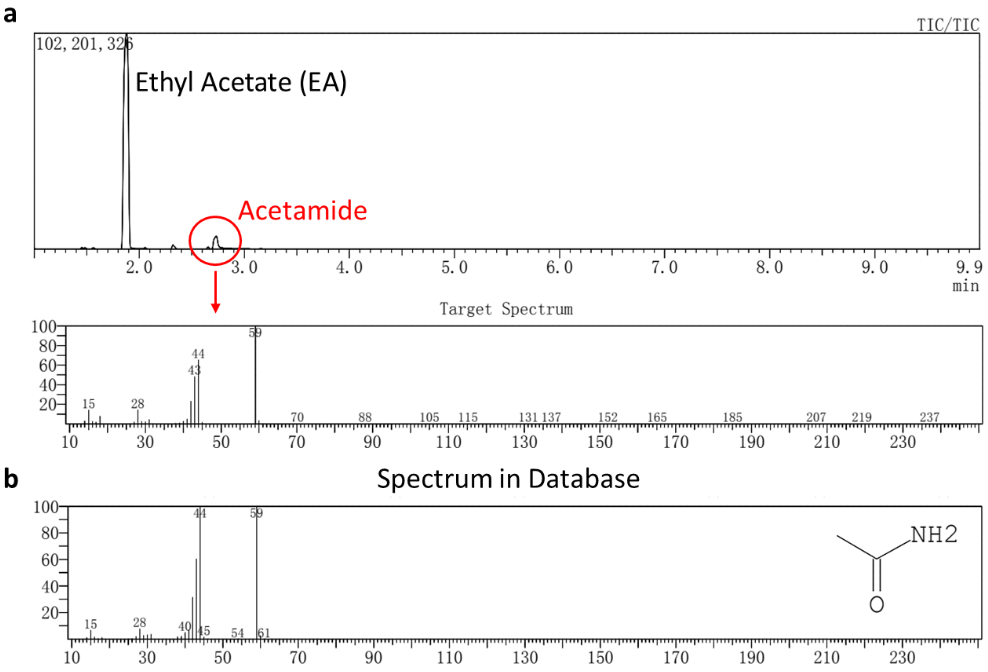

Figure S37. (a) Gas chromatography-Mass spectrometry (SHIMADZU GCMS-QP2020) for purified acetamide (50 mM in ethyl acetate); (b) Standard spectrum of acetamide in the database of GC-MS.

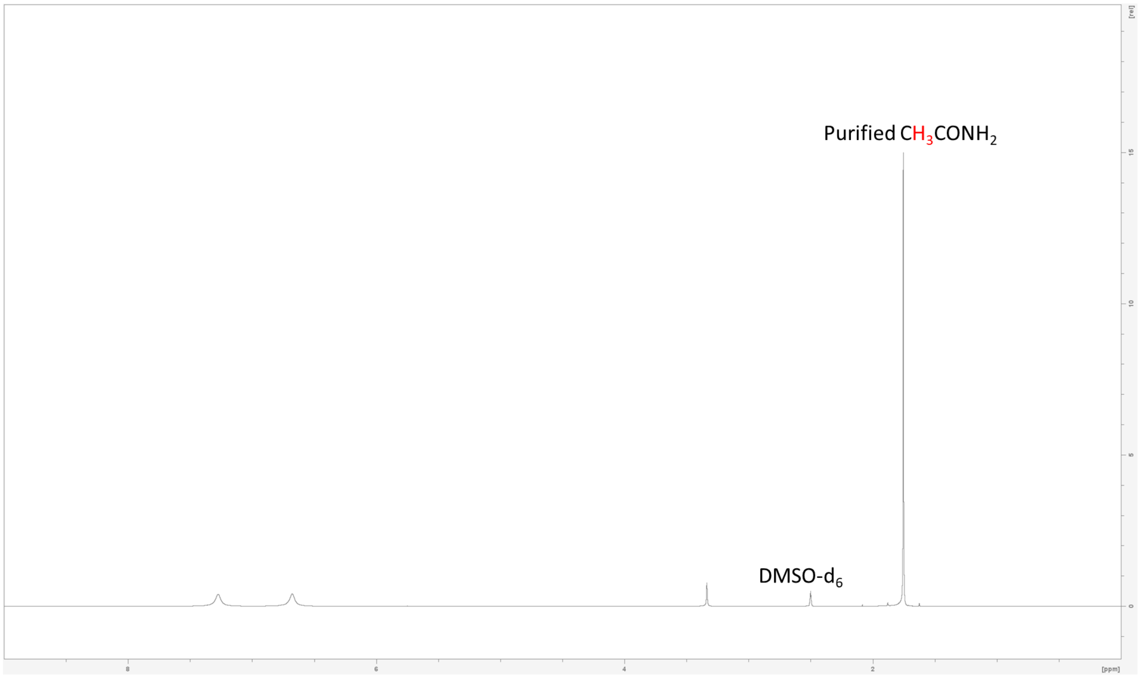

Figure S38. ^1H NMR validation of synthesized and purified acetamide (CH_3CONH_2) after stability test, showing its high purity.

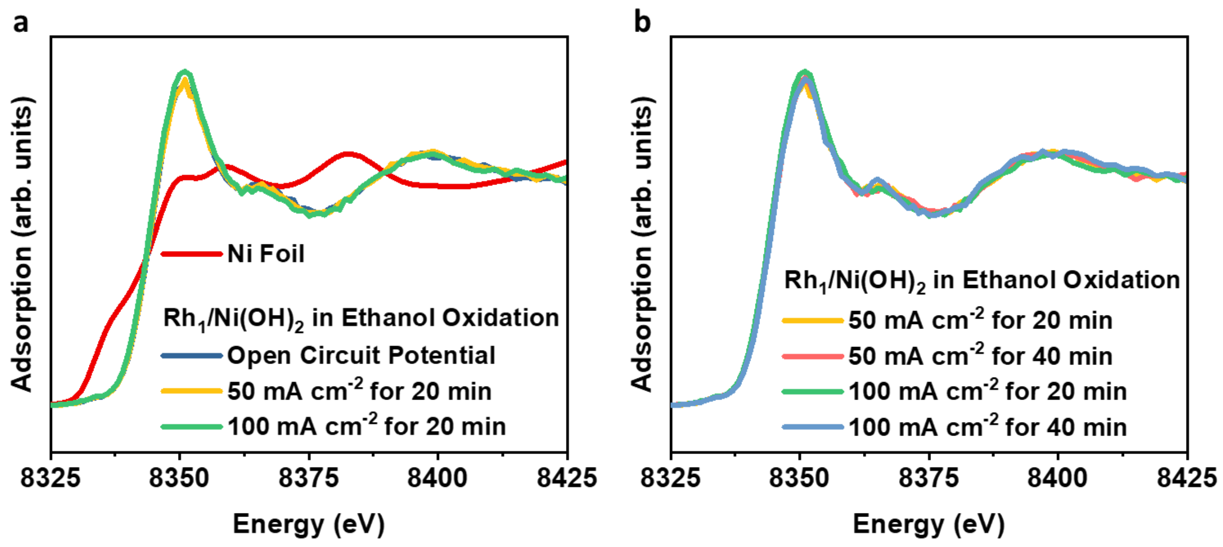

Figure S39. Ni *K*-edge XANES spectra of (a) Ni foil and $Rh_1/Ni(OH)_2$ at different current density in ethanol oxidation; (b) $Rh_1/Ni(OH)_2$ at different current density and different duration time in ethanol oxidation. 1 M KOH and 1 M CH_3CH_2OH was used as electrolyte.

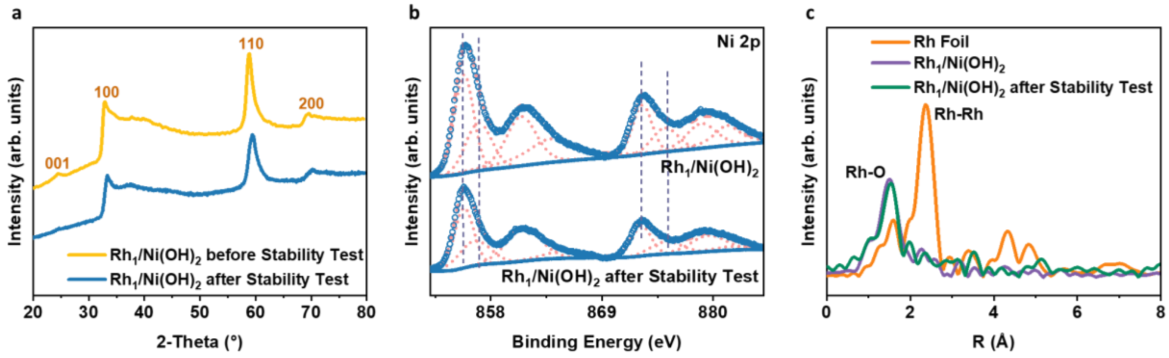

Figure S40. (a) XRD pattern of $Rh_1/Ni(OH)_2$ catalyst, (b) XPS Ni_{2p} core-level spectra of $Rh_1/Ni(OH)_2$ catalyst and (c) Rh *K*-edge EXAFS spectra of $Rh_1/Ni(OH)_2$ catalyst before and after stability test.

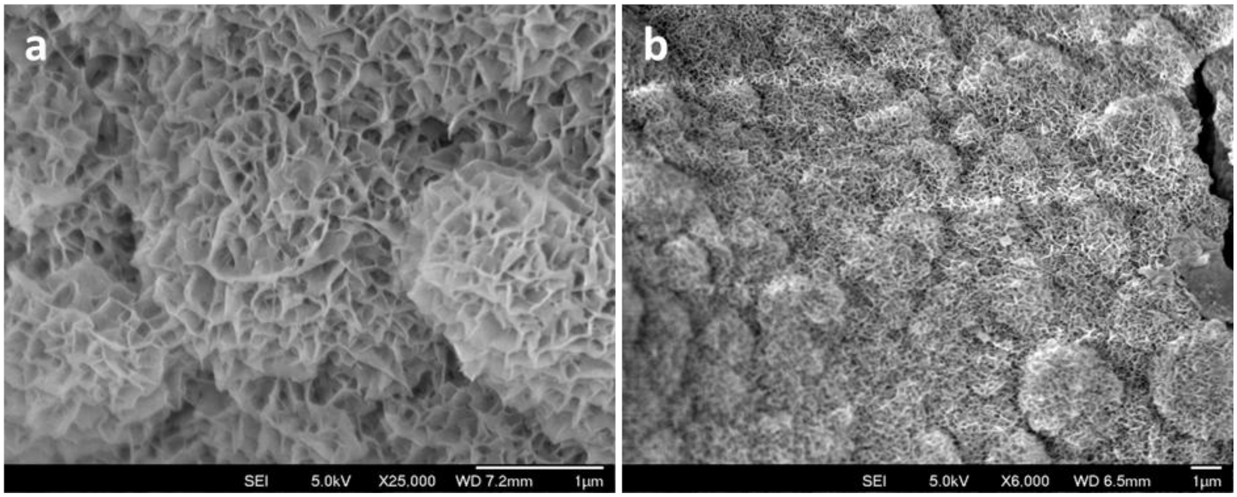

Figure S41. SEM images of $Rh_1/Ni(OH)_2$ catalyst on Ni foam after stability test at (a) 25000x and (b) 6000x magnification.

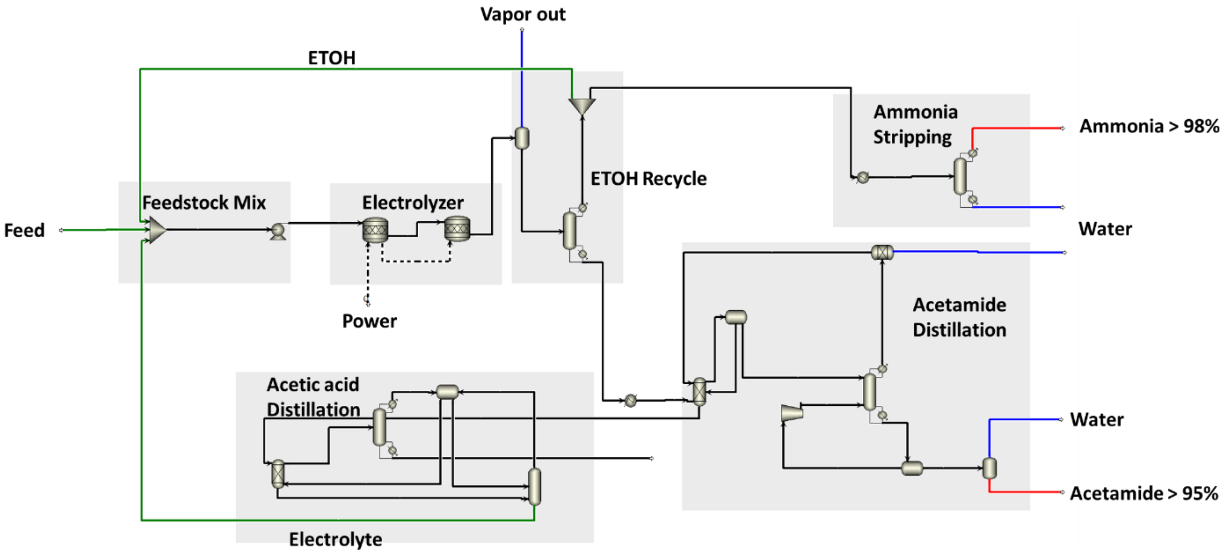

Figure S42. Flowsheet of acetamide electrosynthesis in technical and economic analyses.

Table S1. Elemental analysis of synthesized acetamide.

**Samples**	**C (wt%)**	**H (wt%)**	**N (wt%)**
Synthesized Acetamide	40.41	8.98	23.53
Theoretical Value	40.67	8.53	23.71

Table S2. Comparison of amide electrosynthesis performances with the literature.

Catalyst	Potential	Current	Amide Yield Rate	Ref.
**Ru/Cu_2O||$Rh_1/Ni(OH)_2$**	**2.4 V**	**1.77 A**	**3.5 mmol h^{-1} (0.22 mmol h^{-1} cm^{-2}, formamide)**	**This Work**
**Ru/Cu_2O||$Rh_1/Ni(OH)_2$**	**2.4 V**	**1.73 A**	**7.2 mmol h^{-1} (0.45 mmol h^{-1} cm^{-2}, acetamide)**	**This Work**
**Ru/Cu_2O||$Rh_1/Ni(OH)_2$**	**2.4 V**	**1.38 A**	**3.4 mmol h^{-1} (0.21 mmol h^{-1} cm^{-2}, butyramide)**	**This Work**
Ru_1Cu SAA	-0.5 V *vs.* RHE	~11 mA cm^{-2}	0.09 mmol h^{-1} cm^{-2}, formamide	1
Pt	\	100 mA cm^{-2}	0.305 mmol h^{-1} cm^{-2}, formamide	2
Cu nanoparticles	-1.6 V *vs.* RHE	~800 mA cm^{-2}	~0.2 mmol h^{-1} cm^{-2}, acetamide	3
ox-LIG||Pt foil	-0.58 V *vs.* RHE	~22 mA cm^{-2}	~0.01 mmol h^{-1} cm^{-2}, acetamide	4
Cu NCs/BN	-1.6 V *vs.* RHE	~157 mA cm^{-2}	0.0685 mmol h^{-1} cm^{-2}, acetamide	5

Table S3. Aspen plus modeling parameters for the hybrid extraction-distillation process for acetate and acetamide.

**Parameter**	**Extractor**	**Distillation for Acetate**	**Distillation for Acetamide**	**Stripper for Ammonia**
**P/bar**	1	1	0.5	5
**N_{stages}**	30	30	20	7
**N_{feed}**	1	17	3	1

**Reflux ratio (kmol/kmol)**	0.21	3	3

Table S4. Base case parameter for techno-economic analysis.

Parameter	Unit	Value
**Capital**		
Site preparation	%	10
Contingencies and contractors' fee	%	15
land	%	2
Plant startup	%	2
Computer	USD	20000
**Utility**		
Cooling water	USD per MMGAL	84
Steam@100 PSI	USD per KLB	4.07
PV Electricity	USD per kWh	0.1
Process water	USD per m^3	0.2
Ethyl acetate	USD per kg	1.1
MeOH		0.25
ETOH	USD per kg	0.45
BuOH		0.72
Nitrate	USD per kg	0

Operation		
Direct wages and benefits	USD per hour	35
Direct salaries and benfits	%	15
Operating supplies and services	%	6
Number of workers	-	10
TechAssist to Manufacturing	USD per year	60000
Control lab	USD per year	65000
Shift	-	3
Operating hour	h	8000
**Maintenance**		
Fluid handling process	%	3.5
Salaries and benefits	%	25
Materials and services	%	100
Maintenance overhead	%	5
**Operating overhead**		
General plant overhead	%	7.1
Mechanical department services	%	2.4
Employee relations department	%	5.9

Business	%	7.4
Properties taxes and insurance	%	2
**Depreciation**		
Direct plant	%	8
Allocated plant	%	6
**General Expenses**		
Selling	%	3
Direct research	%	4.8
Allocated research	%	0.5
Administrative expense	%	2
Management incentive compensation	%	1.25
**Products**		
Ammonia	USD per kg	1.2
Formic Acid		0.45
Acetic acid	USD per kg	0.44
Butyric Acid		1.9
Formamide		56.2
Acetamide	USD per kg	50
Butyramide		32.16
**Economic Factors**		
**Plant life**	Year	20
construction period		2
Income tac		38.9

Interest rate		15
MACRS	-	7-years class

Table S5. The upper and lower bound for levelized cost.

Parameters	Unit	Lower bound	Upper bound
**Current density**	mA cm^{-2}	10	200
**Cell voltage**	V	1.4	2.6
**Faradic efficiency (cathode) to acetamide**	%	15	75

Reference

1 Lan, J. *et al.* Efficient electrosynthesis of formamide from carbon monoxide and nitrite on a Ru-dispersed Cu nanocluster catalyst. *Nat. Commun.* **14**, 2870 (2023).

2 Meng, N. *et al.* Electrosynthesis of formamide from methanol and ammonia under ambient conditions. *Nat. Commun.* **13**, 5452 (2022).

3 Kuang, S. *et al.* Acetamide Electrosynthesis from CO(2) and Nitrite in Water. *Angew. Chem. Int. Edit.* **63**, e202316772 (2024).

4 Li, J. & Kornienko, N. Electrochemically driven C-N bond formation from CO(2) and ammonia at the triple-phase boundary. *Chem. Sci.* **13**, 3957-3964 (2022).

5 Wang, Y. *et al.* Atomic-Scale Tailoring C-N Coupling Sites for Efficient Acetamide Electrosynthesis over Cu-Anchored Boron Nitride Nanosheets. *ACS Nano* **18**, 34403-34414 (2024).
