# Supplementary material for: Coupling nitrate electrochemical reduction and nitrite oxidation of ethanol for acetamide synthesis
Source: Nat Commun. 2025 Dec 27;17:1340. doi: 10.1038/s41467-025-68096-3 (PMC12873419; doi:10.1038/s41467-025-68096-3)
Supplement: Supplementary file 2 — Description of Additional Supplementary Files [file 41467_2025_68096_MOESM2_ESM.pdf]

Description of Additional Supplementary Files

File Name: Supplementary Data 1

Description: Atomic coordinates and lattice parameters for optimized of $Rh_1/Ni(OH)_2$ model. This plain text file provides the full crystallographic information for the optimized $Rh_1/Ni(OH)_2$ slab used in our density-functional calculations. It begins with the unit-cell parameters (space group P 1; a = 13.420300 Å, b = 16.238600 Å, c = 18.125099 Å; α = β = γ = 90°; volume = 3949.946187 $Å^3$) followed by a table of fractional atomic coordinates (x, y, z). Entries 1–27 list Ni atoms, 28–81 list H atoms, 82-135 list O atoms, and entry 136 corresponds to the single Rh atom on $Ni(OH)_2$ surface. These coordinates enable exact replication of our computational model.
